# Inherited Variation at *MC1R* and Histological Characteristics of Primary Melanoma

**DOI:** 10.1371/journal.pone.0119920

**Published:** 2015-03-19

**Authors:** Nicholas J. Taylor, Klaus J. Busam, Lynn From, Pamela A. Groben, Hoda Anton-Culver, Anne E. Cust, Colin B. Begg, Terence Dwyer, Richard P. Gallagher, Stephen B. Gruber, Irene Orlow, Stefano Rosso, Nancy E. Thomas, Roberto Zanetti, Timothy R. Rebbeck, Marianne Berwick, Peter A. Kanetsky

**Affiliations:** 1 Department of Cancer Epidemiology, Moffitt Cancer Center, Tampa, Florida, United States of America; 2 Department of Pathology, Memorial Sloan Kettering Cancer Center, New York, New York, United States of America; 3 Women’s College Hospital, Toronto, Ontario, Canada; 4 Departments of Dermatology, Pathology and Laboratory Medicine, Lineberger Comprehensive Cancer Center, University of North Carolina, Chapel Hill, North Carolina, United States of America; 5 Department of Epidemiology, University of California, Irvine, California, United States of America; 6 Sydney School of Public Health, University of Sydney, Sydney, NSW, Australia; 7 Department of Epidemiology and Biostatistics, Memorial Sloan Kettering Cancer Center, New York, New York, United States of America; 8 International Agency for Cancer Research, Lyon, France; 9 British Columbia Cancer Agency, Vancouver, British Columbia, Canada; 10 Keck School of Medicine, University of Southern California Norris Comprehensive Cancer Center, Los Angeles, California, United States of America; 11 Piedmont Tumor Registry, Turin, Italy; 12 Department of Dermatology, Lineberger Comprehensive Cancer Center, University of North Carolina, Chapel Hill, North Carolina, United States of America; 13 Center for Clinical Epidemiology and Biostatistics, Perelman School of Medicine at the University of Pennsylvania, Philadelphia, Pennsylvania, United States of America; 14 Abramson Cancer Center, Perelman School of Medicine at the University of Pennsylvania, Philadelphia, Pennsylvania, United States of America; 15 Departments of Internal Medicine and Dermatology, University of New Mexico, Albuquerque, New Mexico, United States of America; University of Connecticut Health Center, UNITED STATES

## Abstract

Variation in the melanocortin-1receptor (*MC1R*) gene is associated with pigmentary phenotypes and risk of malignant melanoma. Few studies have reported on *MC1R* variation with respect to tumor characteristics, especially clinically important prognostic features. We examined associations between *MC1R* variants and histopathological melanoma characteristics. Study participants were enrolled from nine geographic regions in Australia, Canada, Italy and the United States and were genotyped for *MC1R* variants classified as high-risk [R] (D84E, R142H, R151C, R160W, and D294H, all nonsense and insertion/deletion) or low-risk [r] (all other nonsynonymous) variants. Tissue was available for 2,160 white participants of the Genes, Environment and Melanoma (GEM) Study with a first incident primary melanoma diagnosis, and underwent centralized pathologic review. No statistically significant associations were observed between *MC1R* variants and AJCC established prognostic tumor characteristics: Breslow thickness, presence of mitoses or presence of ulceration. However, *MC1R* was significantly associated with anatomic site of melanoma (p = 0.002) and a positive association was observed between carriage of more than one [R] variant and melanomas arising on the arms (OR = 2.39; 95% CI: 1.40, 4.09). We also observed statistically significant differences between sun-sensitive and sun-resistant individuals with respect to associations between *MC1R* genotype and AJCC prognostic tumor characteristics. Our results suggest inherited variation in *MC1R* may play an influential role in anatomic site presentation of melanomas and may differ with respect to skin pigmentation phenotype.

## Introduction

Inherited variation in the melanocortin-1 receptor (*MC1R*) gene is a robust genetic marker for moderately increased risk of melanoma [1]. We hypothesize that variation in *MC1R* influences the occurrence of melanomas that can be distinguished by histology or other tumor characteristics. However, evidence supporting a consistent association between *MC1R* variation and melanoma tumor characteristics is limited. Direct cross-study comparisons are hindered due in part to a lack of standardized measures and characterization of *MC1R* risk variants [2], coupled with differences in categorization of melanoma characteristics (*e*.*g*. collapsing of anatomic site presentation).

To more thoroughly address whether *MC1R* variants are associated with tumor characteristics, we present results from individuals diagnosed with a first incident primary tumor in a large population-based case-control study of melanoma: the Genes, Environment and Melanoma (GEM) Study. We examined associations between variation in *MC1R* and American Joint Committee on Cancer (AJCC) established tumor characteristics that are associated with prognosis: Breslow thickness and presence of mitoses and ulceration [3–8], as well as with presence of tumor infiltrating lymphocytes (TILs), a purported prognostic factor [9]. We also evaluated other histopathological tumor features for associations with *MC1R* variation in an effort to further characterize potential etiologic heterogeneity.

## Materials and Methods

### GEM Study

The GEM Study is a population-based case-control study that enrolled a large series of individuals diagnosed with a first incident invasive primary cutaneous melanoma. Study participants were identified from eight population-based cancer registries and one hospital center in Australia, Canada, Italy and the United States. Detailed study recruitment methods have been previously described [10,11]. The human research oversight committees at each of the GEM study sites, including those at the British Columbia Cancer Agency, Vancouver, BC, CA; Cancer Care Ontario, Toronto, ON, CA; Centro per la Prevenzione Oncologia, Torino, IT; Memorial Sloan Kettering Cancer Center, New York, NY, US; Menzies Cancer Center, Hobart, TAS, AU; University of California, Irvine, CA, US; University of Michigan, Ann Arbor, MI, US; University of North Carolina, Chapel Hill, NC, US; and University of Sydney, Sydney, NSW, AU, approved the study protocol. Written and signed informed consent was obtained from all participants.

Diagnostic pathology reports were obtained for each participant with a first incident primary melanoma (n = 2,424) from the appropriate ascertainment center, and data corresponding to histological subtype, lesion thickness, and anatomic location of lesion were abstracted. Tumor tissue slides for 2,105 (86.8%) participants with a diagnosis of first incident melanoma were available for centralized pathological review, performed in large part by one of three study pathologists (KB, LF, PG). Standardized pathologic review of slides included evaluation of: histologic subtype, Breslow thickness, Clark level, mitoses, solar elastosis, TILs, presence of satellite lesions, presence of coexisting nevi, presence of pigmentation, evidence of lesion regression, ulceration, and vertical growth phase. Melanomas were classified according to established histopathological criteria [12,13]. Since Breslow thickness was both abstracted from the pathology report and recorded during the centralized pathologic review, the measure corresponding to the deepest reading was chosen to represent the value of most biological relevance.

Using a glossy colored guide to aid in differentiating between nevi and other skin lesions, participants were asked to have the nevi on their backs counted by a family member or friend; logistic models were adjusted for this continuous variable. A phenotypic index was derived using data collected from a study participant self-administered questionnaire [14], and was based on: hair color (black or dark brown = 1; light brown or blond = 2; red = 3), eye color (black or brown = 0; all other colors = 1), and relative inability to tan in response to sun exposure (no = 0; yes = 1) [15]. Phenotypic index scores of 1 and 2 indicate relatively darker cutaneous phenotypes and lower phenotypic melanoma risk; an index score of 3 indicates medium phenotypic risk. Hereinafter, we refer to individuals with any of these three scores as having a “sun-resistant” phenotype. Phenotypic index scores of 4 and 5 indicate relatively fairer cutaneous phenotypes and higher phenotypic risks for melanoma, hereinafter referred to as “sun sensitive”.

### MC1R Genotyping

Details of *MC1R* genotyping methods, distribution of observed *MC1R* variants, and variant carrier status among GEM Study participants have been described previously [15,16]. We adopted nomenclature and definitions based on previous literature [1,17–20] to classify *MC1R* variants as conferring higher risk for melanoma based on strong association with red hair phenotype [R] (D84E, R142H, R151C, R160W, and D294H, all nonsense and insertion/deletion) or lower risk for melanoma based on weaker association with red hair phenotype [r] (all other nonsynonymous variants). Since the exact functional status of many *MC1R* variants is still unknown, we acknowledge that these risk categories may be inaccurate. We categorized *MC1R* carriage into four groups: consensus (absence of any variants), only [r] (carriage of any [r] variant in the absence of a [R] variant), one [R] (carriage of a single [R] variant), and >1 [R] (carriage of more than 1 [R] variant). Secondarily, we examined associations between *MC1R* variant carriage number and tumor characteristics by coding *MC1R* genotype based on total number of variants ([r] and [R]; 0 variants vs. 1 variant vs. 2 or more variants).

### Statistical Analysis

For this report, we include only those GEM participants with first incident primary melanomas who were successfully genotyped for *MC1R* and who self-reported their race as white (n = 2,160). We used SAS (SAS Institute, Cary, NC) to perform multinomial logistic regression to estimate odds ratios (ORs) and 95% confidence intervals (CIs) for the associations between *MC1R* variant status and tumor characteristics while adjusting for sex, age at most recent melanoma diagnosis, study ascertainment center, phenotypic index and number of nevi on the back. For tumor characteristics that were modeled dichotomously, results are equivalent to those obtained from a binomial logistic regression model. We also conducted analyses stratified by sun-resistant and sun-sensitive phenotypes; we then evaluated the Wald p-value of the interaction term for sun sensitivity by *MC1R* to assess heterogeneity of effect between sun-sensitive and sun-resistant phenotypes. All statistical tests were two-sided with an alpha level of 0.05.

## Results

Overall, tumor characteristics were not associated with genotyping success (data not shown). In univariate analyses, we compared the distributions of *MC1R* genotype risk categories across strata of prognostic tumor characteristics including: Breslow thickness and presence of mitoses, ulceration, or TILs. No statistically significant associations were noted among these tumor characteristics. We did observe a statistically significant association between anatomical site and *MC1R* variant carriage based on low-[r] and high-[R] risk variant carriage (p = 0.002) (Table 1). Our findings with respect to *MC1R* variant carriage number were consistent with no association (data not tabulated).

**Table 1:** Adjusted odds ratios (ORs) and 95% confidence intervals (CIs) for the associations between MC1R variants and histopathological tumor characteristics among first incident cases of invasive melanoma in the GEM Study.

		Consensus		Only r^1^		One R^2^		>1 R^2^		Only r^1^ vs. consensus		One R^2^ vs. consensus		>1 R^2^ vs. consensus	
Tumor characteristic		n	%*	n	%*	n	%*	n	%*	OR^3^	95% CI	OR^3^	95% CI	OR^3^	95% CI
**Breslow thickness (mm)**															
	0.01–1.00	251	18	464	33	562	40	136	10	1.00		1.00		1.00	
	1.01–2.00	60	14	141	33	181	43	42	10	1.21	0.85–1.72	1.34	0.95–1.90	1.38	0.83–2.28
	>2.00	49	17	103	33	107	37	32	11	1.12	0.75–1.65	0.94	0.63–1.39	1.02	0.58–1.79
	p = 0.45														
**Mitoses**															
	Absent	173	17	315	32	408	41	96	10	1.00		1.00		1.00	
	Present	120	16	260	35	293	39	81	11	1.06	0.79–1.43	1.01	0.76–1.36	1.16	0.76–1.78
	p = 0.56														
**Ulceration**															
	Absent	267	17	526	33	636	40	158	10	1.00		1.00		1.00	
	Present	26	17	47	31	61	40	18	12	0.81	0.48–1.37	0.99	0.59–1.64	1.38	0.67–2.85
	p = 0.85														
**Tumor infiltrating lymphocytes** ^**†**^															
	Absent	59	15	126	33	156	41	43	11	1.00		1.00		1.00	
	Non-brisk	197	18	367	33	439	39	112	10	0.89	0.61–1.30	0.93	0.64–1.34	1.09	0.63–1.87
	Brisk	34	15	79	34	100	43	21	9	1.07	0.63–1.82	1.09	0.65–1.84	0.93	0.43–2.02
	p = 0.81														
**Anatomic location**															
	Trunk or pelvis	158	17	323	34	402	42	73	8	1.00		1.00		1.00	
	Head or neck	59	18	123	36	131	39	25	7	1.11	0.75–1.63	0.85	0.58–1.25	0.89	0.48–1.65
	Arms	55	14	137	34	157	39	56	14	1.30	0.88–1.92	1.13	0.77–1.66	2.39	1.40–4.09
	Legs	93	20	138	30	174	38	56	12	0.85	0.60–1.22	0.80	0.56–1.13	1.42	0.84–2.41
	p = 0.002														
**Clark level**															
	II	120	17	234	33	294	41	73	10	1.00		1.00		1.00	
	III	94	20	154	32	182	38	48	10	0.79	0.56–1.12	0.74	0.53–1.05	0.72	0.43–1.21
	IV & V	79	15	185	34	222	41	56	10	1.28	0.88–1.86	1.36	0.94–1.96	1.36	0.80–2.30
	p = 0.59														
**Coexisting nevus**															
	None identified	231	18	442	33	524	40	125	10	1.00		1.00		1.00	
	Common acquired	28	12	77	32	102	42	37	15	1.33	0.83–2.14	1.38	0.86–2.19	1.61	0.87–2.97
	Dysplastic	35	17	67	32	89	43	17	8	0.87	0.54–1.38	0.98	0.62–1.54	0.96	0.48–1.93
	Congenital	8	24	11	33	11	33	3	9	0.81	0.30–2.20	0.83	0.30–2.26	0.65	0.14–2.99
	p = 0.16														
**Histological type**															
	Superficial spreading	267	18	472	32	577	40	146	10	1.00		1.00		1.00	
	Nodular	21	12	67	37	74	40	21	12	1.61	0.98–2.74	1.45	0.85–2.45	1.58	0.77–3.26
	Lentigo maligna	34	17	66	33	80	40	18	9	1.16	0.72–1.88	1.11	0.69–1.78	0.91	0.44–1.89
	Not otherwise specified	40	15	98	36	112	42	19	7	1.41	0.91–2.19	1.25	0.81–1.94	0.89	0.45–1.78
	p = 0.28														
**Pigmentation**															
	Present	280	17	545	33	665	40	161	10	1.00		1.00		1.00	
	Absent	22	14	53	33	67	41	21	13	1.23	0.71–2.15	1.17	0.68–2.02	1.37	0.66–2.86
	p = 0.54														
**Regression**															
	Absent	201	16	410	33	488	40	130	11	1.00		1.00		1.00	
	Present	101	17	190	33	241	41	52	9	1.10	0.79–1.54	1.19	0.86–1.65	0.95	0.58–1.56
	p = 0.73														
**Satellite**															
	Absent	199	18	374	33	437	39	113	10	1.00		1.00		1.00	
	Present	1	8	6	50	3	25	2	17	2.30	0.26–20.15	0.77	0.07–8.80	1.36	0.07–26.83
	p = 0.57														
**Solar elastosis**															
	Absent	105	17	206	33	263	42	60	10	1.00		1.00		1.00	
	Present	194	17	378	33	453	40	119	10	1.08	0.76–1.52	1.02	0.73–1.43	1.30	0.79–2.14
	p = 0.79														
**Vertical growth phase**															
	Absent	106	17	207	33	249	40	60	10	1.00		1.00		1.00	
	Present	185	17	364	33	450	40	117	11	1.03	0.75–1.41	1.07	0.79–1.46	1.15	0.73–1.83
	p = 0.77														

* Row percentages are presented

^†^ Potential prognostic factor based on Thomas et al., J Clin Oncology, 2013. Vol. 33, Num. 33: 4252–59

^1^ r indicates carriage of V60L, V92M, I115T, R163Q, or rare nonsynonymous variants in the absence of a R variant.

^2^ R indicates carriage of D84E, R142H, R151C, R160W, D294H, nonsense or insertion/deletion variants.

^3^ ORs are adjusted for center, sex, age at melanoma diagnosis, phenotypic index, and total body mole density

Multivariate analyses are also presented in Table 1. No statistically significant associations were noted among prognostic tumor characteristics. However, our adjusted analyses revealed a strong association between carriage of more than one *MC1R* [R] variant and melanoma development on the arms (OR = 2.39; 95% CI: 1.40, 4.09) when compared to individuals who developed melanomas on the trunk or pelvis. Associations between *MC1R* variants and strata of other melanoma tumor characteristics were consistent with no association after adjustment.

Because previous reports have indicated that melanoma risk associated with carriage of high-risk [R] *MC1R* variants is particularly informative among individuals with darker phenotypic characteristics [21], we explored associations between *MC1R* variants and the four prognostic tumor characteristics by skin pigmentation phenotype. We noted statistical heterogeneity between individuals with sun-sensitive and sun-resistant phenotypes for the associations between *MC1R* variants and Breslow thickness (p = 0.03), presence of mitoses (p = 0.03), presence of ulceration (p = 0.04), as well as presence of TILs (p = 0.01) (Table 2). We observed relatively stronger associations between Breslow thickness and *MC1R* among sun-sensitive individuals. Similarly, we noted pronounced positive associations between carriage of only [r] variants (vs. carriage of only consensus) and presence of mitoses and ulceration among sun-sensitive individuals, whereas carriage of only [r] variants among sun-resistant participants showed little or no association with presence of mitoses and an inverse association with presence of ulceration. We found carriage of [R] variants was more prevalent among sun-sensitive individuals with non-brisk TILs observed in their melanomas compared to sun-resistant individuals with non-brisk TILs. In contrast, both [r] and [R] variants were more prevalent among sun-resistant cases with brisk TILs observed in their lesions compared to sun-sensitive cases with brisk TILs.

**Table 2:** Adjusted odds ratios (ORs) and 95% confidence intervals (CIs) for the associations between MC1R variants and prognostic histopathological tumor characteristics among first incident cases of invasive melanoma in the GEM Study, stratified by phenotype.

		Phenotypically sun-sensitive**										Phenotypically sun-resistant^†^										P_het_
Tumor characteristic		Consensus		Only r^1^		Any R^2^		Only r^1^ vs. consensus		Any R^2^ vs. consensus		Consensus		Only r^1^		Any R^2^		Only r^1^ vs. consensus		Any R^2^ vs. consensus		
		n	%*	n	%*	n	%*	OR^3^	95% CI	OR^3^	95% CI	n	%*	n	%*	n	%*	OR^3^	95% CI	OR^3^	95% CI	
**Breslow thickness (mm)**																						
	0.01–1.00	48	12	87	21	273	67	1.00		1.00		199	21	358	38	391	41	1.00		1.00		0.02
	1.01–2.00	7	6	26	21	88	73	2.05	0.76–5.53	2.38	0.96–5.92	51	18	109	38	126	44	1.10	0.75–1.60	1.18	0.81–1.73	
	>2.00	12	11	37	35	57	54	1.61	0.74–3.50	0.73	0.35–1.52	35	20	61	36	75	44	0.97	0.97–1.54	1.13	0.72–1.78	
	p = 0.01										p = 0.92											
**Mitoses**																						
	Absent	36	12	59	20	199	68	1.00		1.00		133	20	251	38	277	42	1.00		1.00		0.03
	Present	20	8	69	29	151	63	2.03	1.03–4.00	1.31	0.70–2.43	98	20	177	37	207	43	0.91	0.65–1.26	1.05	0.76–1.46	
	p = 0.16										p = 0.99											
**Ulceration**																						
	Absent	54	11	115	23	323	66	1.00		1.00		208	20	397	38	430	42	1.00		1.00		0.04
	Present	2	5	13	32	26	63	3.17	0.67–15.03	1.89	0.42–8.51	23	23	29	28	50	49	0.60	0.33–1.08	0.98	0.57–1.67	
	p = 0.64										p = 0.41											
**Tumor infiltrating lymphocytes** ^**‡**^																						
	Absent	13	11	35	30	75	65	1.00		1.00		44	18	85	35	113	47	1.00		1.00		0.01
	Non-brisk	31	9	72	21	233	69	0.81	0.36–1.84	1.53	0.72–3.24	162	22	285	38	300	40	0.95	0.62–1.45	0.78	0.51–1.18	
	Brisk	12	15	21	26	47	59	0.63	0.23–1.74	0.77	0.30–1.97	22	15	55	38	66	46	1.34	0.71–2.53	1.27	0.69–2.34	
	p = 0.30										p = 0.43											

* Row percentages are presented

** Based on phenotypic index greater than 2

^†^ Based on phenotypic index less than or equal to 2

^‡^ Potential prognostic factor based on Thomas et al., J Clin Oncology, 2013. Vol. 33, Num. 33: 4252–59

^1^ r indicates carriage of V60L, V92M, I115T, R163Q, or rare nonsynonymous variants in the absence of a R variant.

^2^ R indicates carriage of D84E, R142H, R151C, R160W, D294H, nonsense or insertion/deletion variants.

^3^ ORs are adjusted for center, sex, age at melanoma diagnosis, and total body mole density

## Discussion

The GEM Study provides well-annotated histopathological data for melanomas and complete sequencing of participant DNA at the *MC1R* locus, which allows for a comprehensive examination of the associations between variants and histopathological tumor characteristics. In this study we report no pronounced or statistically significant main effect associations of *MC1R* with AJCC accepted prognostic factors of Breslow thickness, presence of ulceration, or presence of mitoses overall. Similarly, we did not observe an association between variation in *MC1R* and TILs, which were shown to be an important independent prognostic feature of melanoma in GEM [9].

However, we did find a persistent positive association between carriage of more than one [R] variant and melanoma presentation on the arms after adjustment. After stratification by skin pigmentation phenotype, this observed association was limited to individuals with sun-resistant phenotypes. There are several previous reports of *MC1R* variation in association with anatomical site of melanoma, but they generally grouped sites together on the basis of sun-exposure before analyses and/or categorized *MC1R* variants differently, [22–26] and are not directly comparable to this study. We did attempt to draw a comparison between our results and results from a case-control study of sporadic and familial melanoma in a Swedish population [25], which reported an increased association between carriage of ≥1 *MC1R* variant and melanoma presentation on the trunk (OR = 1.54; 95% CI: 1.01, 2.37). After recapitulating their coding for anatomic site, *MC1R*, and other covariates to the best of our ability, we were unable to replicate that finding (data not shown). Recently, Peña-Vilabelda *et*. *al*. reported results similar to our own with respect to high-risk *MC1R* variants in association with melanoma tumor site presentation (Arms: OR = 2.34; 95% CI: 0.98, 5.61) [27]. Interestingly, prior studies have noted more favorable prognoses among melanomas presenting on the extremities [28,29]. Future studies of variation in *MC1R* related to anatomical melanoma presentation are necessary to validate our findings.

We explored effect modification by phenotypic index only among the prognostic measures of Breslow thickness, ulceration, mitoses, and TILs to limit the potential for false discovery. We observed significant differences between phenotypically sun-resistant and sun-sensitive individuals with respect to all four prognostic tumor factors. Interestingly, sun-sensitive cases demonstrated stronger associations across Breslow thickness, mitoses and ulceration compared to those observed among sun-resistant individuals. These results are thought-provoking considering that it is among individuals with more sun-resistant phenotypes that *MC1R* has been associated with increased risk for melanoma [21,30]. However, we did note generally stronger associations between brisk TILs and *MC1R* among individuals with a sun-resistant phenotype compared to sun-sensitive cases. Although associations between *MC1R* variant carriage and all four prognostic variables were significantly different between phenotypic classifications, we were likely underpowered to detect associations within strata of phenotypic index despite the large sample size available in the GEM Study.

This investigation of tumor characteristics among 2,160 first incident cases of melanoma is the largest such study to examine associations with germline variation in *MC1R*. A strength of this study is the population-based nature of the parent GEM Study, from which a large number of incident cases were drawn from nine international ascertainment centers, improving generalizability of results to persons of European ancestry living in a variety of climates. Other advantages of this investigation were the centralized histopathological review conducted by expert pathologists and the ability to adjust for the potential impact of skin pigment and number of nevi. However, we do acknowledge the possibility that false positive findings may have arisen due to multiple hypothesis testing and the exploratory nature of associations examined between *MC1R* variation and tumor factors stratified by phenotypic index; thus, these findings should be validated in larger study populations before more meaningful interpretations can be made.
